# The MRI characteristics of the no-flow region are similar in reperfused and non-reperfused myocardial infarcts: an MRI and histopathology study in swine

**DOI:** 10.1186/s41747-017-0001-x

**Published:** 2017-06-29

**Authors:** Gabriel A. Elgavish, Tamas Simor, Rob J. van der Geest, Pal Suranyi, Pal P. Kiss, Zsofia Lenkey, Robert Kirschner, Dezhi Wang, Brigitta C. Brott, Akos Varga-Szemes

**Affiliations:** 1 Department of Biochemistry and Molecular Genetics, University of Alabama at Birmingham, MCLM 556, Birmingham, AL 35294-0005 USA; 2 Division of Cardiovascular Disease, Department of Medicine, University of Alabama at Birmingham, FOT 907, Birmingham, AL 35294-3407 USA; 3 Elgavish Paramagnetics Inc., 1737 Valpar Dr, Hoover, AL 35226 USA; 4 Heart Institute, Medical School, University of Pecs, Szigeti ut 12, Pecs, 7624 Hungary; 5 Department of Radiology, Leiden University Medical Center, Albinusdreef 2, Leiden, 2333 ZA The Netherlands; 6 Division of Cardiovascular Imaging, Department of Radiology and Radiological Science, Medical University of South Carolina, 25 Courtenay Dr, Charleston, SC 29425 USA; 7 Department of Pathology, Histomorphometry and Molecular Analysis Core, University of Alabama at Birmingham, LHRB 589A, Birmingham, AL 35294-0007 USA

**Keywords:** Myocardial infarction, Microvascular obstruction, No-flow region, Magnetic resonance imaging (MRI), Late gadolinium enhancement

## Abstract

**Background:**

The no-flow region (NF) visualised by magnetic resonance imaging (MRI) in myocardial infarction (MI) has been explained as the product of reperfusion-injury-induced microvascular obstruction. However, a similar MRI phenomenon occurs in non-reperfused MI. Accordingly, our purpose was to compare the MRI and histopathologic characteristics of the NF in reperfused and non-reperfused MIs.

**Methods:**

Reperfused (*n* = 7) and non-reperfused MIs (*n* = 7) were generated in swine by percutaneous balloon occlusion and microsphere embolisation techniques. Four days post-MI, animals underwent myocardial T2-mapping, early and serial late gadolinium enhancement MRI. MI and NF were compared between the models using the independent samples *t* test. Serial measurements were analysed using repeated measures analysis of variance. Triphenyltetrazolium chloride (TTC) macroscopic and microscopic histopathologic assessment was also performed.

**Results:**

The MI size in the reperfused and non-reperfused groups was 17.1 ± 3.4 ml and 19.4 ± 8.1 ml, respectively (*p* = 0.090), in agreement with TTC assessment (*p* = 0.216; *p* = 0.484), and the NF size was 7.7 ± 2.4 ml and 8.1 ± 1.9 ml, respectively (*P* = 0.211). Compared to the reference 2-min post-contrast measurement, the NF size was significantly reduced at 20 min in the reperfused group and at 25 min in the non-reperfused group (both *p* < 0.001). Nevertheless, the NF was still detectable at 45 min after injection. No significant T2 difference was observed between the groups (*p* > 0.326). Histopathologic assessment revealed extensive calcification and hemosiderin deposition in the NF of the reperfused MI, but not in the non-reperfused MI.

**Conclusions:**

The NF in non-reperfused and reperfused MIs have similar characteristics on MRI despite the different pathophysiologic and underlying histopathologic conditions, indicating that the presence of the NF alone cannot differentiate between these two types of MI.

## Key points

- The no-flow region (NF) is characteristic of both reperfused and non-reperfused myocardial infarct (MI)
- The size of the NF is significantly reduced in the post-contrast period
- The relative size of the NF and the uptake of contrast in the NF are similar in both models
- There is excessive calcification and haemorrhage in the NF in reperfused MI, but not in non-reperfused MI

## Background

The clinical management of myocardial infarction (MI) by early reperfusion therapy aims to reduce the extent of the ischaemic injury and to prevent the long-term consequences of MI [1]. However, Braunwald and Kloner assert that reperfusion may also result in reperfusion injury, i.e., the death of potentially salvageable myocardium [1, 2]. Reperfusion injury has been defined as reperfusion-related expansion of ischaemic cardiac injury resulting in decreased contractility, increased arrhythmogenic effects, irreversible conversion of reversible myocyte injury, and microvascular obstruction (MO) [1, 3].

The presence of MO seems to play a major role in long-term outcome after reperfusion post MI [4–11]. MO has been described as the strongest predictor of increase over time in left ventricle (LV) end-diastolic and end-systolic volumes post MI [4]. Patients with MO have been shown to have a significantly larger MI than patients without MO [5]. MO detected one week after the onset of MI has been suggested as an independent predictor of the size of MI at one year follow up and was found to be associated with adverse infarct healing, adverse LV remodelling, increased LV volumes, and lower ejection fractions [10]. Patients with MO have been reported as having more cardiovascular events than those without it [12]. Even independently of MI size, the presence of MO may remain a prognostic marker of post-MI complications [11]. Thus, as mentioned previously, the presence and/or the size of the MO might be one of the strong prognostic parameters after reperfusion post MI [4, 5, 8–11, 13].

MO has been defined as a product of reperfusion injury [1]. It has been shown, however, that the very same magnetic resonance imaging (MRI) phenomenon, i.e. central hypo-enhancement within the infarcted area, also occurs in MI without reperfusion [14, 15]. In non-reperfused MI, with no possibility of reperfusion injury, the underlying pathophysiologic condition of the similar MRI phenomenon might be different. Thus, to prevent any confusion when referring to the unenhanced central area within the MI observed by MRI, we use the term “no-flow region” (NF) instead of “microvascular obstruction”. In this study, we aimed to compare the MRI and histopathologic characteristics of the NF in non-reperfused and reperfused MI.

## Methods

### Animal models

The study protocol was approved by the Institutional Animal Care and Use Committee and complied with the Guidelines for the Care and Use of Laboratory Animals (National Institutes of Health). Male swine (*n* = 14, weight 24 ± 3.1 kg) were anesthetised with an intramuscularly administered mixture of telazol (4.4 mg/kg) and xylazine (4.4 mg/kg). Following intubation, animals were ventilated mechanically (Model 2000, Hallowell EMC, Pittsfield MA, USA) and anaesthesia was maintained by continuous administration of isoflurane (2.0–2.5% V/V). Normal body temperature was supported using a heating pad. Heart rate and blood oxygen saturation were monitored, and electrocardiogram was recorded.

The right femoral artery was surgically prepared and cannulated using a 6-F arterial sheath (Pinnacle, Terumo Medical Co, Elkton, MD, USA). Heparin (100 IU/kg) was administered intravenously and the activated clotting time was monitored and adjusted as needed with additional heparin to maintain the activated clotting time above 300 s. A 6-F coronary guide catheter (RunWay Kimny Mini, Boston Scientific, Natick MA, USA) was introduced to cannulate the ostium of the left main coronary artery, and initial coronary angiography (Philips BV Pulsera, Best, The Netherlands) was performed.

### Reperfused myocardial infarct model

In animals assigned to the reperfused group (*n* = 7), a 2.0–2.5 mm angioplasty balloon (Maverick, Boston Scientific, Natick MA, USA) was introduced over a coronary guide wire into the left circumflex (LCX) coronary artery. After determining the proper balloon position for the occlusion, the balloon catheter was inflated and left in position for 90 min to induce MI. After 90 min of ischaemia the balloon was deflated and removed, and coronary angiography was repeated to confirm recanalisation. The femoral artery was decannulated and surgically ligated, and the wound was closed.

### Non-reperfused myocardial infarct model

In animals assigned to the non-reperfused group (*n* = 7), the microsphere embolisation technique was used [15]. Briefly, a 2.9-F straight tip microcatheter (Merit Maestro, Merit Medical, South Jordan UT, USA) was introduced over a coronary guide wire into the LCX coronary artery. After determining the proper microcatheter position for the occlusion, using the radiopaque tip of the microcatheter, the coronary guide wire was pulled out and a mixture of 900-μm microspheres (Embozene, CeloNova BioSciences, Inc., Newnan GA, USA) was flushed into the coronary artery under fluoroscopic control. When the lumen of the coronary artery distal to the tip of the microcatheter was completely filled with microspheres, the injection was stopped, the occlusion was confirmed by repeated angiography, and the microcatheter was removed. At the end of a 90-min monitoring period, the occlusion was confirmed by repeated angiography, and the femoral artery was decannulated and surgically ligated, and the wound was closed.

### Magnetic resonance imaging

**Fig. 1:**
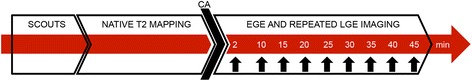
Flowchart of the protocol for magnetic resonance imaging (MRI). *Vertical black arrows* indicate early gadolinium enhancement (*EGE*) and late gadolinium enhancement (*LGE*) acquisitions. CA, contrast agent

MRI studies were carried out four days after the induction of MI, using a 1.5 T GE Signa-Horizon CV/i scanner (GE Healthcare, Milwaukee, WI) equipped with a cardiac phased-array coil. Pigs were anaesthetised and ventilated mechanically as described previously. Imaging was performed during “breath-hold” at end-inspiration using the following parameters: field of view = 300 mm, image-matrix = 256^2^, and slice thickness = 10 mm. The flowchart of the MRI protocol is shown in Fig. 1.

### T2 mapping

Following the pilot scans, short-axis oriented T2 mapping was carried out to study the T2 characteristics, especially the presence of myocardial haemorrhage in the MI. T2 maps were generated employing a double inversion recovery (IR), fast spin-echo pulse sequence using the following parameters: flip angle (α) = 90°, echo train length = 24, and echo time (TE) = 12–105 ms (12, 20, 30, 45, 60, 75, 90, and 105 ms). Images were collected at end-diastole in every second cardiac cycle.

### Early and late gadolinium enhancement imaging

Following T2 mapping, a bolus of 0.2 mmol/kg gadopentetate dimeglumine (Magnevist, Bayer HealthCare Pharmaceuticals Inc, Wayne NJ, USA) was administered as the contrast agent (CA) for early gadolinium enhancement (EGE) and late gadolinium enhancement (LGE) imaging. Segmented, 180°-prepared, IR fast-gradient-echo, short-axis (6 to 8 short-axis slices to cover the entire LV from apex to base) and long-axis (two-chamber, four-chamber, and LV outflow tract) oriented images were generated 2 min after CA administration (EGE imaging), and the acquisition was repeated at 10 min and thereafter every 5 min ending at 45 min (totaling eight LGE acquisitions in each animal). Imaging parameters were: α = 25°, TE = 3.2 ms, views per segment = 16, and repetition time (TR) = 5.5 ms. The inversion time (TI) for EGE images was 500 ms. For the LGE images, the applicable TI was set and continuously adjusted to an optimum value for nulling the signal of the healthy myocardium.

At the end of the planned in vivo MRI session, the animals were killed using a mixture of pentobarbital sodium, propylene glycol and ethyl alcohol (0.2 ml/kg) (Fatal-Plus®, Vortech Pharmaceuticals, Dearborn MI, USA). Euthanasia was ascertained by electrocardiogram and auscultation above the thorax. The hearts were excised after euthanasia, rinsed with saline, and prepared for further studies.

### Magnetic resonance image analysis

The Research Mass cardiovascular MR evaluation software (Leiden University Medical Center, Leiden, The Netherlands) was used for image analysis. The endocardial and epicardial contours of the LV were traced manually in every series of the short-axis images to delineate the myocardial area. Myocardial pixels were counted, and based on the pixel dimensions, the myocardial volume of each slice was determined, from which the total LV myocardial mass (LVM, g) was also calculated using the specific gravity of 1.05 g/ml of myocardial tissue. To avoid observer bias, instead of manual contouring, a thresholding technique was used to delineate the MI and NF.

**Fig. 2:**
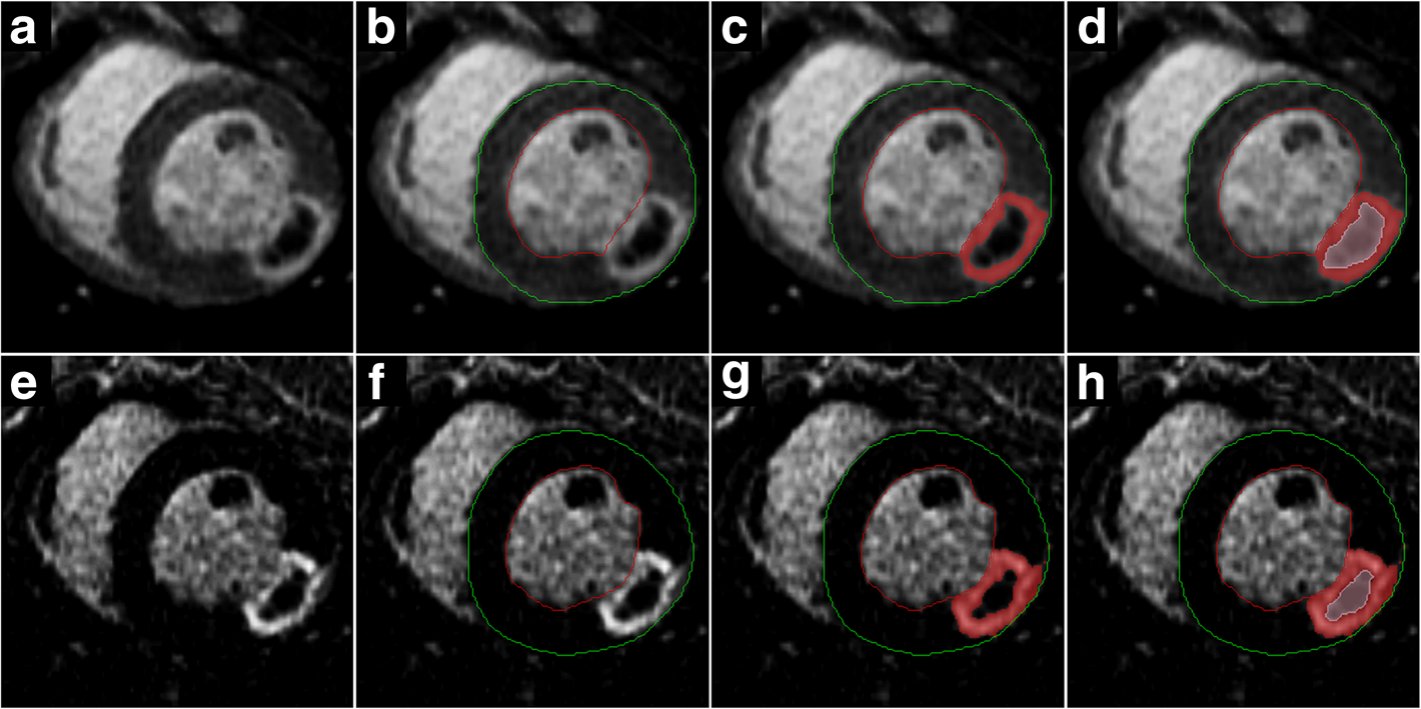
Myocardial infarct (MI) and no-flow region (NF) segmentation. Short-axis inversion recovery (IR) early gadolinium enhancement (EGE) (**a**-**d**) and late gadolinium enhancement (LGE) (**e**-**h**) images obtained from an animal that underwent induction of non-reperfused MI. The presence of the central NF surrounded by the rim of the MI is apparent in the raw images (**a**, **e**). After the endocardial (*red*) and epicardial (*green*) contours were traced (**b**, **f**), the enhanced area was determined by the thresholding method using remote mean + 5 SD as the cutoff (**c**, **g**). To measure the NF, the central non-enhanced area was traced using a dedicated contour (**d**, **h**). The total MI size was calculated as the sum of the enhanced and the NF areas. The NF size was measured on the EGE image (**d**) and the total MI size was assessed in the corresponding LGE image (**h**)

The mean signal intensity (SI) of the normal myocardium was measured using a region of interest containing at least 100 pixels. The mean SI of the remote myocardium plus five times the standard deviation (SD) of this mean was used as a threshold to delineate the pixels within the infarct (“infarcted pixels”) (Fig. 2) [16, 17]. Pixels with SI over this threshold value were considered “MI pixels”.

As the NF has been defined as a hypoperfused, unenhanced core within the highlighted MI, the SI of the NF is equal to or less than the SI of the remote myocardium. To overcome the problem of the NF not showing enhancement in EGE (the time period when its size is the largest), the size of the NF was measured indirectly. After MI thresholding, the unenhanced area of the NF was included in the MI area using the “MVO” contour function of the Mass application (Fig. 2c). Combination of the highlighted MI area and the unenhanced NF area showed the true, total MI area. The difference between the total and the highlighted areas provided the NF area (Fig. 2d). The volume of the MI and NF were each expressed as a percentage of the LVM: the MI fraction (MIF) was calculated as the MI volume/LVM % and the NF fraction (NFF) was calculated as the NF volume/LVM %. NF was also expressed as a percentage of the volume of the MI. Change in the size of the NF over time during the post-contrast period was analysed using the aforementioned technique. The actual size of the NF area was determined at each post-contrast time point.

### T2 calculation

T2 was calculated from the TE dependence of the SI by means of a two-parameter, least squares, curve-fitting routine, using the following formula:

$$ \mathrm{S}\mathrm{I} = {\mathrm{SI}}_0 \times {\mathrm{e}}^{\left(-\mathrm{TE} \times \frac{1}{\mathrm{T}2}\right)} $$

where SI_0_ is the signal intensity at the theoretical TE = 0 time point and it also represents the maximum SI.

The segmentation of the MI was carried out based on the areas determined in the EGE images. The T2 of the remote, infarcted (showing LGE), and the NF myocardium were obtained.

### Histopathologic assessment

#### Triphenyltetrazolium-chloride staining

Triphenyltetrazolium-chloride (TTC) staining was used as a post-mortem reference standard to confirm the existence and the size of the MI. The hearts were bread-sliced using a commercial meat slicer, and subsequently the slices were incubated for 20 min with a buffered (pH 7.4) 1.5% TTC solution at 37 °C, similar to the method as described by Fishbein et al. [18]. After staining, the slices were immersed in 10% formalin for 20 min to increase the contrast between the healthy and the infarcted myocardium. Finally, both surfaces of each slice were digitally scanned with an image scanner, and both sides of each slice were analysed using ImageJ (Wayne Rasband, NIH, USA). The LV myocardium and the MI area were manually contoured and quantified. LVM and MIF were determined in each heart.

#### Microscopic histologic assessment

After TTC, whole-heart slices, including the defined epicardial and endocardial borders, were submitted for histopathologic assessment. The samples were fixed in 10% formalin, embedded in paraffin, and sectioned at 5-μm thickness. Haematoxylin-eosin staining (for general evaluation), Prussian blue staining (for haemorrhage detection), and Von Kossa staining (for calcium detection) were performed. Histologic samples were evaluated using a histomorphometry system (BioQuant Image Analysis, Nashville TN, USA) equipped with an Olympus BX-51 video microscope (Olympus America, Center Valley PA, USA).

### Statistical analysis

Statistical analysis of the data obtained was carried out using MedCalc 13.2.2 (MedCalc Software, Ostend, Belgium). The Kolmogorov-Smirnov test was used to confirm that the data had a Gaussian distribution. T2 measurements and infarct-related parameters (MI and NF volume, MIF, and NFF) were compared in the reperfused and non-reperfused models using the independent samples *t* test, and MI size and LVM measured by MRI and TTC were compared using the paired samples *t* test. The size of the NF area was measured at each post-contrast time point and repeated measures analysis of variance was used to determine the time when the size of the NF was significantly different from the reference EGE measurement. Results were reported as mean ± standard deviation (SD), unless otherwise noted. *p* values lower than 0.050 were considered significant.

## Results

**Fig. 3:**
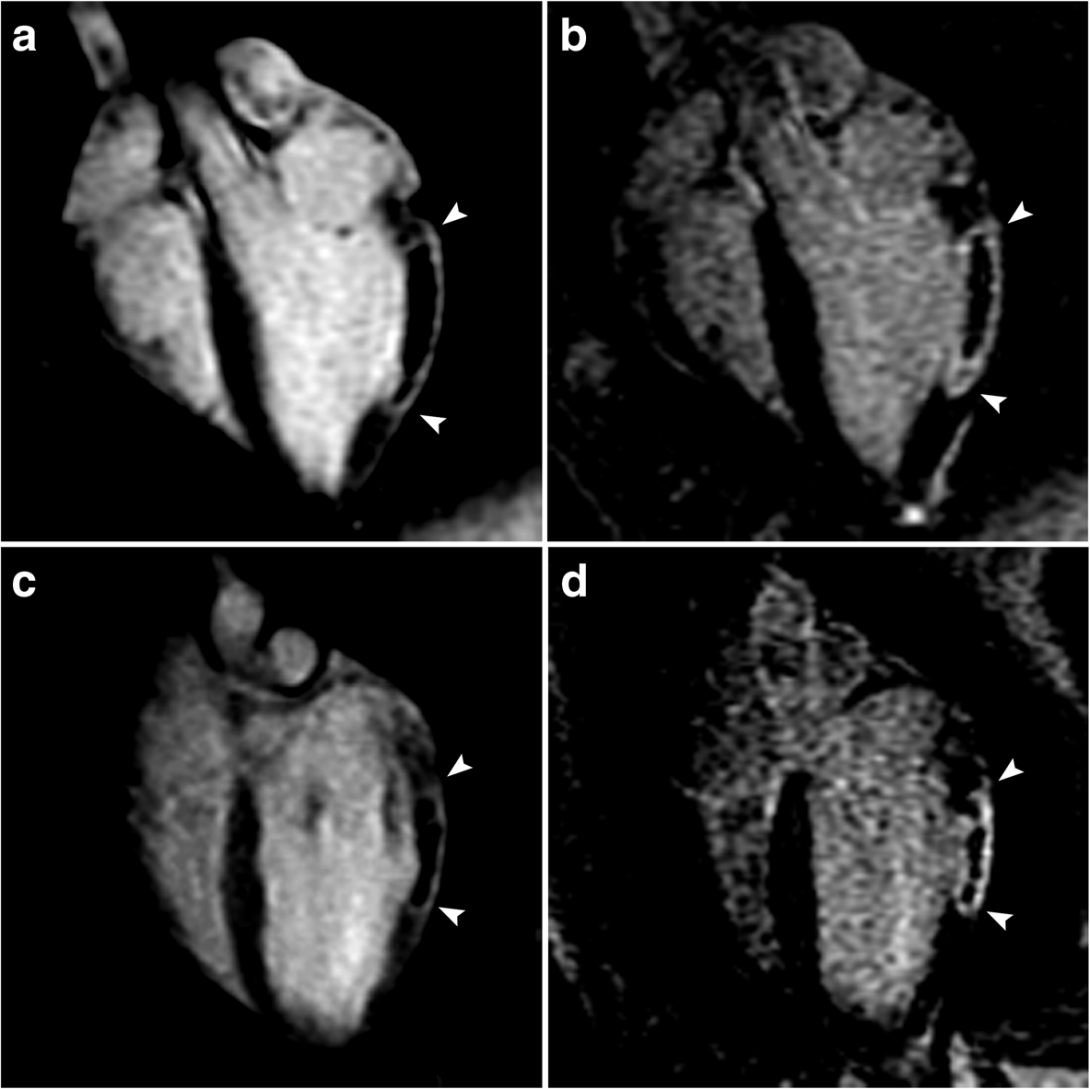
Magnetic resonance imaging (MRI) appearance of myocardial infarct (MI) with a significant no-flow region (NF) at post-contrast time points that are used clinically. Representative long-axis early gadolinium enhancement (EGE) (2 min post-contrast) (**a**, **c**) and late gadolinium enhancement (LGE) (15 min post-contrast) (**b**, **d**) images obtained from animals with reperfused MI (**a**, **b**) (*top row*) and non-reperfused (**c**, **d**) (*bottom row*). MIs are bracketed by *arrowheads*. The NF can be clearly visualised as a central unenhanced area within the enhanced rim of the MI, even in the late phase in both models

All animals included in this study survived until day 4 and underwent MRI. The NF was detected in all 14 swine involved in this study. Representative long-axis EGE images (performed 2 min after CA administration) and LGE images (performed 15 min after CA administration) from both experimental groups are shown in Fig. 3.

LVM, MI, and NF measurements obtained by MRI and TTC are shown in Table 1. There was good agreement between MRI and TTC for the measurement of average LVM in each model (all *p* > 0.077). There were no significant differences in mean MI size measured by MRI versus TTC, either in the reperfused group (*p* = 0.216), or in the non-reperfused group (*p* = 0.484). Furthermore, there was no significant difference in the mean MI size in the reperfused and non-reperfused groups (*p* = 0.090). The mean NF size, and its values normalised as a percentage of MI or LVM measured by EGE MRI, was not significantly different in the two experimental groups (all *p* > 0.114).

**Table 1:** Average LVM, MI size (including NF) and NF size, as well as their normalised values (mean ± SD) obtained by MRI and TTC

Parameter	Reperfused			Non-Reperfused			*P* ^*^
	MRI	TTC	*P*	MRI	TTC	*P*	
LVM (g)	69.4 ± 21.3	74.2 ± 27.9	0.094	80.2 ± 22.3	76.6 ± 21.7	0.109	0.077
MI (ml)	17.1 ± 3.4	18.8 ± 4.4	0.216	19.4 ± 8.1	18.1 ± 7.8	0.484	0.090
MIF (%LVM)	27.3 ± 12.2	30.5 ± 15.5	0.202	24.3 ± 7.7	24.6 ± 10.1	0.625	0.158
NF (ml)	7.7 ± 2.4			8.1 ± 1.9			0.211
NFF (%LVM)	12.6 ± 7.8			10.1 ± 3.3			0.114
NFF (%MI)	44.6 ± 7.6			41.5 ± 9.9			0.278

*MRI* magnetic resonance imaging, *TTC* triphenyltetrazolium chloride staining, *LVM* left ventricular mass, *MI* myocardial infarct, *MIF* MI fraction, *NF* no-flow region, *NFF* no-flow fraction. ^*^ *P* value for comparison of measurements in the reperfused and non-reperfused groups based on MRI

T2 relaxation times measured in the remote, MI, and NF area are reported in Table 2. No significant difference was observed between the groups, or between the remote and the NF area. T2 was significantly increased in the infarct area, i.e., the area showing LGE at 15 min post CA injection, in comparison to both the remote and NF areas (all *P* < 0.001).

**Table 2:** Native T2 relaxation times (ms, mean ± SD)

	Remote	Infarct	No-flow region
Reperfused	57 ± 5	94 ± 11^*^	56 ± 14
Non-Reperfused	63 ± 7	96 ± 17^*^	60 ± 9
*p*	0.326	0.765	0.432

^*^Significant difference (*p* < 0.001) compared to the remote myocardium

**Fig. 4:**
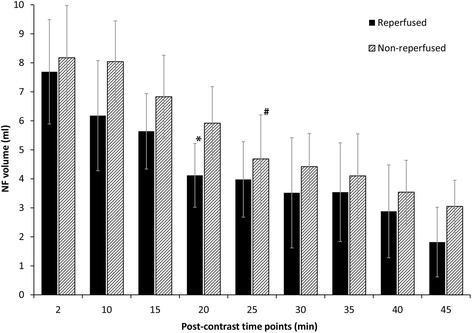
Change over time in the no-flow region (*NF*) volume after contrast agent injection in reperfused and non-reperfused myocardial infarct. The first NF measurement performed at 2 min after contrast agent injection by early gadolinium enhancement imaging was considered the reference. *Significant reduction in the NF volume compared to the reference was observed at 20 min and thereafter in the reperfused model; ^#^significant reduction in the NF volume compared to the reference at 25 min and thereafter in the non-reperfused model

**Fig. 5:**
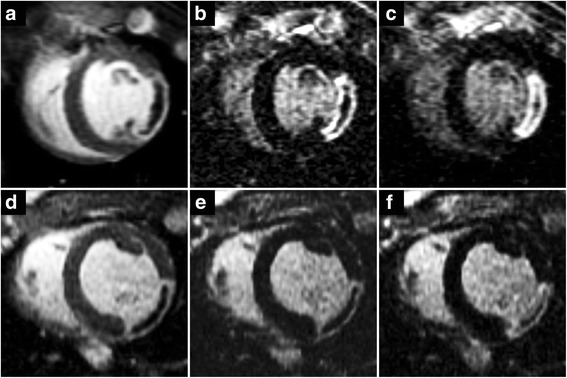
Contrast uptake characteristics of the no-flow region (NF) in reperfused and non-reperfused myocardial infarct (MI). Short-axis oriented post-contrast inversion recovery images acquired in a reperfused (*top row*) and a non-reperfused (*bottom row*) animal at 2 min (**a**, **d**), 15 min (**b**, **e**), and 45 min (**c**, **f**) after the administration of the contrast agent (CA). Early gadolinium enhancement images (**a**, **d**) are best to visualise the NF, while late gadolinium enhancement images at 15 min post contrast (**b**, **e**) are regularly used clinically to highlight MI. The area of the NF is the largest immediately after CA administration and gradually shrinks over time. By the end of the imaging session 45 min post contrast (**c**, **f**) the area previously shown as the NF now seems to be partially highlighted. CA distribution within the NF, however, is not uniform and the NF still contains unenhanced areas

The volume of the NF gradually decreased with time after CA administration in both models (Fig. 4). NF measurements taken at and after 20 min were significantly different from the size of NF obtained in EGE in the reperfused model (*P* < 0.001), and there was a similar, significant reduction in the NF at 25 min in the non-reperfused model (*P* < 0.001). The NF was still observed in both models at 45 min after CA administration. The size of the NF, however, was larger in the non-reperfused group (3.05 ± 1.20 versus 1.82 ± 0.91 ml, respectively, *p* < 0.001), indicating different CA uptake kinetics in the two models. The size of the MI (the enhanced and the NF area combined) did not change significantly during the imaging period in either of the two models. Representative images including both EGE and LGE images are shown in Fig. 5.

**Fig. 6:**
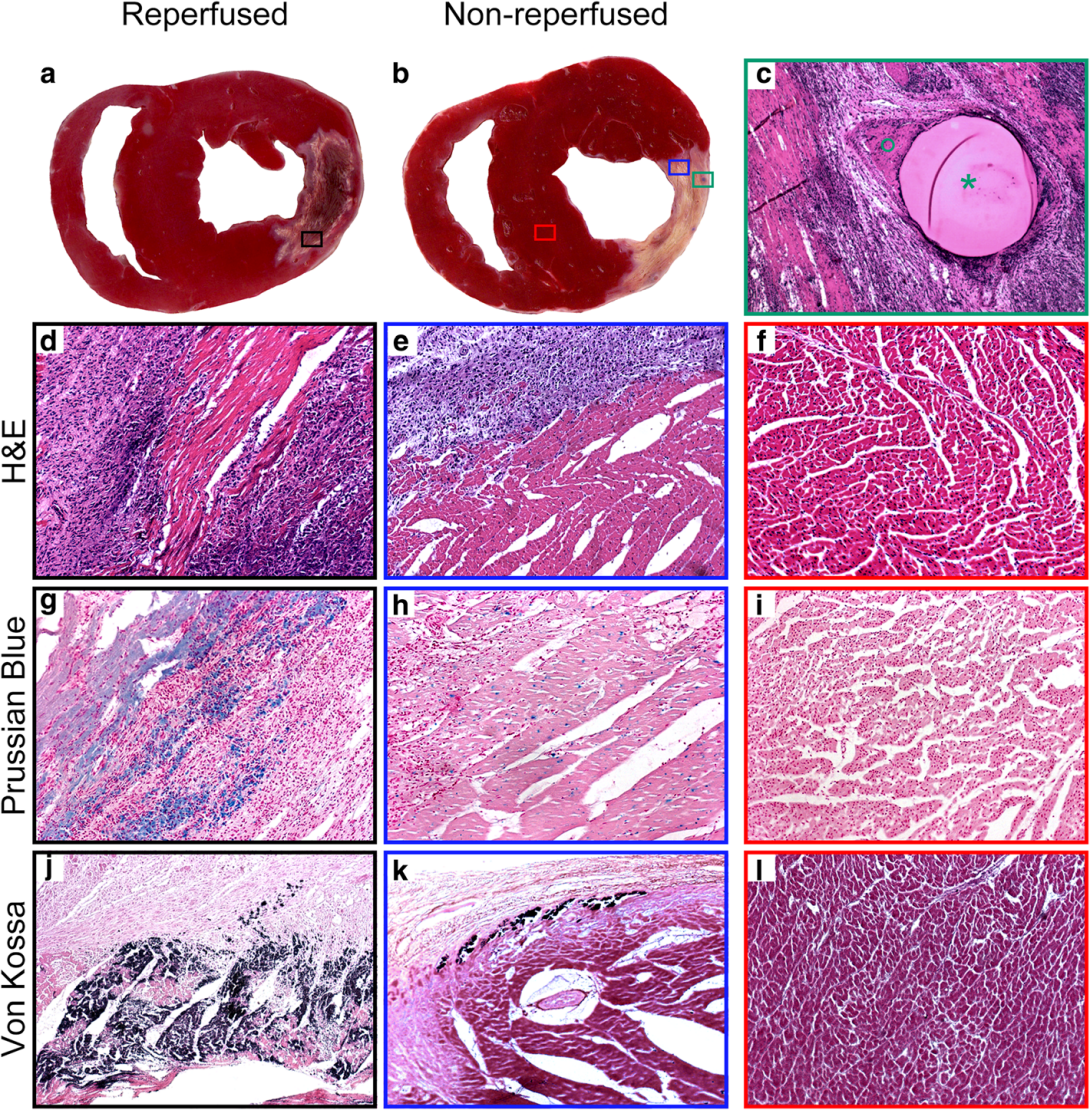
Histopathologic evaluation of reperfused and non-reperfused myocardial infarct (MI). Representative triphenyltetrazolium-chloride (TTC)-stained heart slices and histopathologic samples are shown. TTC slices were prepared from the same animals as shown previously (see Fig. 4) and the slices correspond to the magnetic resonance imaging (MRI) slices shown above. The visual TTC assessment of the MI in the reperfused model (**a**) shows a remarkable central brown region indicating the presence of a significant amount of blood (formaldehyde turns blood brown). TTC in the non-reperfused model (**b**), however, shows the entire MI as a nearly homogenous unstained area. The microspheres used for generating the occlusion in the non-reperfused model can be clearly seen by the naked eye (**b**) (*green rectangle*). The microsphere (**c**) (*green star*) (H&E × 10 magnification) is entrapped in a small epicardial branch of the left circumflex (LCX) coronary artery and the remaining lumen of the vessel is filled with thrombi and blood cell remnants (**c**) (*green circle*). MI regions were further evaluated using H&E (**d**, **e**) (×20 magnification), Prussian blue (**g**, **h**) (×20 magnification), and Von Kossa (**j**, **k**) (×20 magnification) staining; *black rectangles* and *blue rectangles* indicate the sampled regions as shown in **a**, **b**, respectively). Details are provided in Results. Normal myocardial tissue (*red rectangle* shows sampling region (**b**)) stained using H&E, Prussian blue and Von Kossa staining (**f**, **i**, **l**) is displayed for comparison

Macroscopic and microscopic histopathologic evaluation of representative reperfused and non-reperfused MI is shown in Fig. 6. In the haematoxylin-eosin slides, widespread myocyte necrosis (nuclear shrinkage and nuclear loss), oedema (indicated by wide interstitium), and inflammatory cell infiltration (polymorphonuclear, considered neutrophils) and mononuclear (considered lymphocytes and macrophages) were observed in the central MI regions (corresponding to MRI NF areas) in both models. Using Prussian blue and Von Kossa staining, notable differences were detected between the reperfused and non-reperfused model. In reperfused MI, Prussian blue showed a relevant amount of iron deposits (extensive blue spots) throughout the whole MI area, but at the highest concentration in the border zone of the MI. Iron deposits were not detected at all in the core area of the non-reperfused MI. However, some iron-related blue spots were observed sporadically in the border zone of the non-reperfused MI. In reperfused MI, Von Kossa staining suggested that the periphery of the MI was strongly positive for calcium, while the inner core was negative. Calcium deposits were observed in the border zone of the non-reperfused MI as well, but the amount of the calcium was clearly smaller and less widespread than in the reperfused MI.

## Discussion

In this study we qualitatively investigated the MRI CA uptake characteristics of the NF area in non-reperfused and reperfused MI on day 4 after experimental induction of MI. Based on previous experience [15], we expected emergence of an NF area in the majority of the non-reperfused animals. The literature indicates that the NF is detected in human reperfused MI in around 68% of patients [19] and our past experience of experiments in reperfused swine suggests that the NF can be expected in at least two thirds of animals. In this study, all reperfused animals presented with a significant NF on day 4 post MI.

Our study indicates that the NF size strongly depends on the time elapsed after the administration of the CA. Of note, with time the NF was able to take up the CA even in animals with non-reperfused MI. According to the current definition, the NF should be measured during the early CA kinetics [20]. Due to the significant CA uptake into the NF, this protocol should indeed be followed, as evaluating the NF 20–25 min later in the post-contrast period would result in notably larger enhanced MI regions and smaller NF areas. As previous reports highlight the prognostic value of the NF, its accurate quantification is even more relevant [21].

The reason for CA uptake in the reperfused animals may be explained by the observation that ischaemia/reperfusion injury does not really cause total occlusion of the microvascular system [1]. Apparently, blood flow in the area of the early NF is not completely absent but rather very low [1]. The NF first appears centrally in the infarct core extending toward the epicardium over time. Myocardial blood flow progressively decreases within 2 to 3 hours after reperfusion, resulting in a twofold increase in the area of the NF. A further increase in NF size has been demonstrated up to 48 hours after initiation of reperfusion [1]. In contrast, myocardial blood flow in non-reperfused MI is completely absent. Nevertheless, as our results indicate, the NF area in non-reperfused MI also takes up CA, which has also been reported in prior studies.

Wang et al. have shown that the NF in non-reperfused MIs can be detected by EGE and LGE imaging up to 22–32 min after CA administration. It then disappears as the area defined as the NF on EGE images becomes enhanced [14]. Our study partially supports this observation, but it also indicates that the NF in non-reperfused MI can be detected beyond approximately 30 min and even at 45 min after the administration of CA.

Assuming total coronary artery occlusion in the non-reperfused MI model, the only possible mechanism for CA uptake is slow interstitial diffusion from the surrounding tissues that are still well-supplied [14]. The rim of the MI is highlighted even as early as the time of the EGE acquisition, presumably by CA from intact collaterals. This rim area of the MI is usually composed of a mixture of viable and nonviable myocytes [22]. The collateral circulation supplying this peri-infarct territory keeps the myocardial tissue partially alive, and serves as a CA source as well. The peri-infarct area, however, also contains vessels with damaged endothelial lining, allowing the accumulation of the CA in the extracellular space. This extracellular space may serve as a pool to supply the adjacent infarct area, including the NF, with CA. Interestingly, the reperfused and the non-reperfused MIs accumulate CA at a similar rate. The latter observation may confirm the hypothesis that most of the CA uptake is through slow interstitial diffusion in both MI models. If the blood flow is not completely absent in the reperfused case, as suggested by others [20], a vascular component may contribute to the access to CA. This flow, however, may be extremely limited, as the rate of contrast uptake in both models seems similar.

In this project we studied the presence of hemosiderin components by T2 relaxation rate measurements. Although T2* mapping is the most widely used technique for the detection of haemorrhage, T2 mapping has also been established for such assessment [23]. Our results indicate that T2 measured in the NF was not significantly different from the T2 observed in the remote myocardium and also did not differ between the models. Although this observation seems unexpected given that our histopathologic studies showed extensive hemosiderin deposition in the reperfused but not in the non-reperfused NF, it might be explained by the possible overlap of oedema (causing T2 increase) and haemorrhage (causing T2 decrease) resulting in an apparent net zero T2 change compared to normal tissue in the reperfused model. On the other hand, an absence of both oedema and haemorrhage would also result in an unchanged T2 in the non-reperfused model.

Another histopathologic difference between the two models was the presence of calcium deposits in the NF, which may also contribute to T2 shortening. Calcium deposition is a component of reperfusion injury [24], and its presence only in the reperfused animals further supports the successful total occlusion of the coronary arteries in the non-reperfused animals.

### Limitations

The major limitation of this study is that the pulse sequence used to image the MI only allowed the qualitative assessment of the contrast uptake in the NF area. To quantitatively evaluate the diffusion-related parameters to characterise the contrast uptake, fast T1 mapping sequences would be needed, as the speed of the conventional T1 mapping protocols is insufficient to monitor such diffusion changes. Novel modified Look-Locker inversion recovery (MOLLI)-based pulse sequences [25] would allow for single-shot, single-breath-hold T1 mapping with high spatial and temporal resolution. However, such MOLLI pulse sequences were not available on the MRI scanner utilised for this study. In addition, post-contrast T1 mapping would have allowed the calculation of extracellular volume fraction providing additional details on myocardial tissue characterization. Post-contrast T1 mapping without a MOLLI sequence, however, would have required an extensive acquisition time, interfering with our study design, which required post-contrast imaging in 5-min intervals.

Another minor limitation is that the presented MRI data were obtained 4 days after induction of MI; thus, our results are not necessarily applicable to MI assessed at different time points. Also, the animal models used in this study may not be fully comparable to human MI. Thus, our results cannot be directly translated to human myocardial tissue characterization. Finally, the number of animals in this project was small but the study was still sufficiently powered with this cohort.

## Conclusions

NF in non-reperfused and reperfused MIs have similar characteristics on MRI despite the different pathophysiologic and underlying histopathologic conditions, indicating that the presence of the NF alone cannot differentiate between these two types of MI.

## Abbreviations

CA: contrast agent
EGE: Early gadolinium enhancement
H&E: Haematoxylin and eosin
IR: Inversion recovery
LCX: Left circumflex coronary artery
LGE: Late gadolinium enhancement
LV: Left ventricle
LVM: Left ventricular mass
MI: Myocardial infarct
MIF: Myocardial infarct fraction
MO: Microvascular obstruction
MRI: Magnetic resonance imaging
NF: No-flow region
NFF: No-flow region fraction
SD: Standard deviation
SI: Signal intensity
TE: Echo time
TR: Repetition time
TTC: Triphenyltetrazolium-chloride
